# Isolation of *Allocrenothrix methanica* reveals distinct ecophysiologies of filamentous methanotrophs and adaptations to O_2_ limitation

**DOI:** 10.1093/ismejo/wrag178

**Published:** 2026-07-03

**Authors:** Kazuhiro Umezawa, Jackson Makoto Tsuji, Yukinori Tani, Seichi Nohara, Rudolf I Amann, Manabu Fukui

**Affiliations:** School of Food and Nutritional Sciences, University of Shizuoka, Shizuoka, 422-8526, Japan; Institute for Extra-cutting-edge Science and Technology Avant-garde Research, Japan Agency for Marine-Earth Science and Technology, Yokosuka, 237-0061, Japan; Division of Environmental Photobiology, National Institute for Basic Biology, Okazaki, 444-8585, Japan; School of Food and Nutritional Sciences, University of Shizuoka, Shizuoka, 422-8526, Japan; Fukushima Oze Conservation and Research Association, Fukushima, 960-8670, Japan; Department of Molecular Ecology, Max Planck Institute for Marine Microbiology, Bremen, 28359, Germany; The Institute of Low Temperature Science, Hokkaido University, Sapporo, 060-0819, Japan

**Keywords:** methane, methanotrophs, freshwater, wetlands, redox, genomics, filamentous, Crenothrix, Allocrenothrix

## Abstract

Ferdinand Cohn observed abundant filamentous bacteria in drinking water wells in 1870 that he named *Crenothrix polyspora*. Subsequent research has revealed the methanotrophic metabolism of Crenothrix bacteria and their disproportionately high activity in stratified lakes compared to unicellular methanotrophs, yet laboratory cultivation has proven elusive, leaving the ecophysiology of Crenothrix bacteria largely unknown. Here we report the isolation of two methanotrophic strains of the “lacustrine Crenothrix” clade from an iron-rich wetland and reveal their unique cell biology and ecology. We demonstrate that the strains are microaerobic and grow as filaments of cells, which are connected by unidirectionally oriented structures. The strains have broad genomic repertoires for addressing O_2_ limitation that are uniquely associated with lacustrine Crenothrix compared to related clades based on genome data. Aligning with laboratory observations, we identify lacustrine Crenothrix bacteria along potential redox gradients in the wetland at iron-rich snow sites, and we also detect such bacteria in diverse global ecosystems based on public metagenome searches. Together, our data strongly point to an ecophysiology of lacustrine Crenothrix bacteria that is tightly linked to O_2_ limitation, and we propose that the strains uniquely store or share metabolic intermediates between cells in filaments to thrive under such conditions. Our cultivation-based findings for these strains, which we name *Allocrenothrix methanica*, provide new insights into the diversity, evolution, and ecology of filamentous methanotrophs, connecting over 150 years of microbiology research and opening vast new opportunities to investigate bacteria contributing to the global methane cycle under O_2_ limitation.

## Introduction

Methane is a potent greenhouse gas whose emissions are increasing globally from anthropogenic sources and aquatic ecosystems such as wetlands, lakes, and oceans [1, 2]. In freshwater systems, which produce methane equivalent to a quarter of the terrestrial carbon sink [3], aerobic methane-oxidizing bacteria (methanotrophs) that rely on molecular oxygen (O_2_) as a reactant in their metabolism play a crucial role in suppressing methane emissions [4–6]. Yet how these methanotrophic bacteria respond to environmental changes, such as increasing oxygen depletion of water bodies [7, 8], is unclear, leaving a large knowledge gap for how methane emissions may change or can be managed [5, 9].

Due to the production of methane in anoxic regions, aerobic methanotrophs are commonly found at the boundaries of where O_2_ becomes available in ecosystems. To survive in such O_2_-depleted settings and maximize their methane access, methanotrophs require adaptations to manage O_2_ limitation. Previous work has reported the use of alternate metabolisms such as denitrification [10] or fermentation [11] by methanotrophs to reduce their O_2_ demands, partnership of methanotrophs with oxygenic phototrophs to utilize cryptic O_2_ [12], and motility of methanotrophs to reach regions with optimal O_2_ levels [13] (reviewed in [9]). With the exception of some recent studies (e.g. [14, 15]), such work has generally focused on free-living unicellular methanotrophic bacteria, which are routinely studied in laboratory culture, despite the occurrence of morphologically diverse forms, such as filamentous methanotrophs, in the environment.

Ferdinand Cohn described filamentous bacteria in drinking water wells over 150 years ago at the dawn of modern microbiology [16]. Although initially suspected to metabolize iron [17], the described species, named *Crenothrix polyspora*, was later confirmed to be methanotrophic using molecular approaches [18]. Subsequent work has detected such filamentous Crenothrix bacteria not only in drinking water and groundwater systems but also in stratified lakes, where they made disproportionately high contributions to methane oxidation compared to unicellular methanotrophs [14]. Other characterized filamentous bacteria employ unique strategies compared to free-living microorganisms to address O_2_ limitation, including sheath-based gliding motility to access nutrients [19, 20], storage of metabolic substrates in vacuoles for use under different redox conditions [21, 22], and transport of electrons along filaments to metabolize across redox gradients [23, 24]. Crenothrix bacteria may thus have distinct ecophysiologies and modes for metabolizing methane compared to their unicellular relatives, with important implications for the microbial methane cycle. Yet despite over a century of microbiology research, Crenothrix bacteria have proven elusive to stably cultivate or isolate in the laboratory, leaving the environmental role of these bacteria and their physiologies severely understudied.

In this study, we report the isolation of two filamentous methanotroph strains from the “lacustrine Crenothrix” clade and provide a detailed description of their cell biology, physiology, ecology, and taxonomy. By combining growth experiments with comparative genomics and field studies, we reveal the unique ecophysiology of these filamentous methanotrophs, their association with redox boundaries, and their adaptations that are in contrast with those reported for unicellular strains. Our work provides important insights into an environmentally relevant form of methanotrophic bacteria and opens crucial new avenues to understand the ecophysiological drivers of the microbial methane cycle.

## Materials and methods

### Isolation of filamentous methanotrophs

Iron-rich microbial mat material was sampled from Oze National Park (Japan) at a wetland site in the park’s western region (site KOJ02; 36°55.025′ N, 139°11.888′ E) in June and August 2022. Following sampling, mat material was inoculated into custom basal cultivation media (see Supplementary Methods and Table S1) in glass serum bottles with a micro-oxic (~5% O_2_) or anoxic headspace. Strains were isolated by serial dilution and sorting of cells using a micromanipulator (Supplementary Methods). Purity was checked by inoculation onto agar plates with 10 times diluted R2A medium.

### Amplicon and genome sequencing

Cells grown in PSVn medium (developed in this study; headspace of 63.3: 2: 16.7: 16.7 dinitrogen: carbon dioxide: methane: air at 1.5 atm pressure) were harvested from turbid cultures by centrifugation. Extraction of DNA from harvested cells was performed using the Wizard Genomic DNA purification Kit (Promega, Madison, WI). Extracted DNA was used to perform amplicon sequencing of the 16S rRNA gene (V4 region) using a MiSeq System (Illumina, San Diego, CA) as described in the Supplementary Methods. The same DNA samples were then used for whole genome sequencing. Sequencing libraries for long-read sequencing were prepared using the Native Barcoding Kit 24 V14 (SQK-NBD114.24; Oxford Nanopore Technologies, Didcot, UK), and resulting libraries were each sequenced on three Flongle flow cells (R10.4.1) for 24 h. Short-read sequencing was performed by the Bioengineering Lab Co. (Sagamihara, Japan) using the DNBSEQ-T7 platform (2x150 bp reads; see Supplementary Methods).

Amplicon data was analyzed via QIIME2 version 2024.2 [25, 26], including denoising by DADA2, and hybrid assembly of genome sequencing data was performed using the Hybracter pipeline [27] version 0.7.4 (Supplementary Methods). After assembly, genomes were annotated using DFAST version 1.6.0 [28], and functional genes were predicted using METABOLIC-G version 4.0 [29] and FeGenie version 1.0 [30]. Taxonomic classification was performed using GTDB-Tk version 2.4.0 based on Genome Taxonomy Database (GTDB) release 220 [31, 32].

### Physiological analyses

Methane and O_2_ concentrations in culture headspaces were measured over time using a gas chromatograph with a thermal conductivity detector (GC3210S; GL science, Tokyo, Japan) and WG-100 column (GL science). Cultures were grown in PSVn medium under a methane-containing micro-oxic headspace (as above) with 0.5 mM poorly crystalline ferric oxide particles (estimated molar iron concentration; see Supplementary Methods) and 0.1 μM lanthanum(III) nitrate. During analysis, helium was used as the carrier gas (46 ml min^−1^), the oven and injector were heated to 50°C, and the detector was used with an electric current of 120 mA at 55°C.

For transmission and scanning electron microscopy analyses, isolates were grown in PSVn medium as above, with the exception that lanthanum was omitted, until cultures were turbid. Harvested cells were then fixed and prepared for analysis as described in the Supplementary Methods, with sample preparation and observation being performed by Tokai Electron Microscopy, Inc. (Nagoya, Japan). Samples for transmission and scanning electron microscopy were observed under a JEM-1400Plus transmission electron microscope (JEOL, Tokyo, Japan) and JSM-7500F scanning electron microscope (JEOL), respectively.

We additionally performed time-lapse observations of the strain AF98 culture during growth. The culture was inoculated in PSVn medium (without ferric oxide particles but with 0.1 μM lanthanum(III) nitrate) on a glass Petri dish, and the petri dish was placed into a custom gas-tight glass chamber. A gas mixture (50: 25: 25 of dinitrogen: methane: air) was continuously provided at 10 ml min^−1^ to the chamber headspace, and the chamber was incubated at 20°C–25°C under an inverted microscope (IX71; Evident, Tokyo, Japan). Phase-contrast images of growing cells were taken every 30 s.

Maximum cell densities of typical cultures were determined by cell counting under an optical microscope. Before microscopy, bundles of filaments were separated by gentle shearing using a 25G needle and syringe. Approximate doubling times of cultures were calculated based on typical incubation times required to reach turbidity and known inoculum volumes from parent cultures.

### Comparative genomics and phylogenetics

Genome sequences of selected members of the *Methylomonadaceae* family were obtained from the National Center for Biotechnology Information database together with the genome of an outgroup *Methylococcales* member. (GTDB-based taxonomy is used throughout this work; *Methylomonadaceae* corresponds to the *Methylococcaceae* family in taxonomy described under the International Code of Nomenclature of Prokaryotes.) The reference set was selected to have broad taxonomic coverage within the family while retaining genomes with high completeness (minimum 95%) and low contamination (maximum 5%) based on CheckM2 for each taxon [33]. Additional representatives were retained from the UBA4132 and *Crenothrix* genera, which were the focus of this study. The resulting genome set, including strains AF98 and MI19235, contained 37 genomes (or 38 including the outgroup strain). A phylogenomic tree was then built using GToTree version 1.8.3 [34] based on 170 concatenated marker proteins as described in the Supplementary Methods. A 16S rRNA gene phylogeny was constructed to complement this analysis. Sequences were aligned using MAFFT version 6.864, and a maximum likelihood phylogenetic tree was built, based on 1260 conserved nucleotide sites, using the T3p + G + I evolutionary model with 100 bootstrap iterations via MEGA11 [35–37]. Selected environmental 16S rRNA gene sequences from public databases, including those classified as *Crenothrix* in the Silva database (release 138.2) [38], were included in an extended phylogenetic analysis. The sequences were aligned using MAFFT and used to construct a maximum likelihood phylogeny via MEGA12 [39] with 1189 conserved nucleotide sites. The TN93 + G + I evolutionary model was used with 1000 bootstrap iterations.

The 37 genomes and outgroup genome were annotated using METABOLIC-G version 4.0 [29] and FeGenie version 1.0 [30]. In addition, whole protein-coding genes in the genomes were classified into homologous groups using OrthoFinder version 2.5.5 [40] for further gene identification. Potential bacteriohemerythrins and hemerythrin domain-containing proteins were identified based on a conserved protein domain family for hemerythrin (ID cl15774) using the Batch Web version of the CD-search tool [41]. Methanol dehydrogenases XoxF and MxaF were identified by phylogenetic placement. Sequences were aligned via MAFFT with a previously published reference sequence set [42], and aligned sequences were used to construct a maximum likelihood phylogenetic tree, based on 500 conserved amino acid sites, using MEGA11 with 100 bootstrap iterations and the LG + G evolutionary model. To identify genes encoding nitrite reductases (*nirS* and *nirK*), a profile Hidden Markov Model set described previously [43] was used to search predicted protein sequences via hmmsearch version 3.4 with an e-value threshold of 10^−100^ [44].

Orthologous gene groups were further compared between genomes via an ordination. Counts of orthologous groups per genome were normalized as proportions within each genome. Singleton orthologous groups (i.e. with only one gene member across all genomes) were excluded from calculations. The resulting proportion table was used to calculate a dissimilarity matrix via the diversity.beta_diversity function in scikit-bio version 0.6.2 [45] using Bray–Curtis dissimilarities. The stats.ordination.pcoa function in scikit-bio was then used to generate the ordination by principal coordinate analysis (PCoA). Groupings of genomes by clade were tested for statistical significance using a permutational analysis of variance (PERMANOVA). The stats.distance.permanova function of scikit-bio was used with 10 000 permutations.

To complement analyses of the 37 genome reference set, expanded sets of 99 and 313 genomes associated with the *Methylococcales* order were also downloaded and analyzed to perform a pan-core analysis and semi-automated searches of denitrification genes and hemerythrin domain-containing genes. Analysis is described in the Supplementary Methods.

### Collection of environmental samples

To understand the distribution of filamentous methanotrophs in the environment and their potential activity, we surveyed the wetland site across summer, fall, and spring of 2022 and 2024. In summer and fall, we targeted sites with robust iron mats, and we compared these to sites having iron material mixed with plant matter (“iron residue” sites) and sites outside the iron-rich zone of the park (“non-iron” sites). In spring, we sampled snow overlying iron-rich sites and took either snow cores or surficial snow samples where snow was in the final stages of spring melt. Samples for DNA extraction were collected as bulk material or by filtration and were preserved with DNA/RNA Shield (Zymo Research; California, USA; details in Supplementary Methods). Samples were then kept cool until return to the laboratory, where they were frozen. For analyses of iron-rich snow cores using catalyzed reporter deposition fluorescence in situ hybridization (CARD-FISH), material was collected in sterile bags, allowed to melt, and transported cool to the laboratory for fixation. Collection of physicochemical parameters, including total dissolved iron concentrations, is described in the Supplementary Methods.

### Amplicon sequencing of environmental samples

Extraction of DNA was performed using the collected environmental material or filter membranes using the ZymoBIOMICS DNA Miniprep Kit (D4300; Zymo). Amplicon sequencing of the 16S rRNA gene (V3–V4 region) was then performed for the samples by the Bioengineering Lab Co. (Sagamihara, Japan; see Supplementary Methods). QIIME2 version 2024.10 [25] was used to analyse the amplicon sequencing data, including primer trimming, sequence denoising, and taxonomic classification based on the Silva r138 SSU database [38]. Sequences were further classified into potential *Crenothrix* clades by phylogenetic placement as described in the Supplementary Methods.

An ordination was constructed for the sequenced environmental samples. Samples were rarefied to the lowest sequence count value in the dataset (i.e. 26 686) using the feature-table rarefy module of QIIME2. Following this, scikit-bio version 0.6.2 [45] was used to construct the ordination as a PCoA distance biplot as described in the Supplementary Methods. A PERMANOVA was performed, to test if grouping samples by wetland site type was statistically robust, using the same methods as for comparative genomics data above.

Relative abundances of *Methylococcales* members were compared between iron mat samples and other wetland samples using scipy version 1.16.1 [46] in Python version 3.12.4. A single representative sample from each site and sampling season was selected for the test (see Supplementary Data 2). Given that the assumption of normality was violated based on the scipy stats.normaltest function, a Mann–Whiteney *U* test was used for the comparison via the stats.mannwhitneyu function.

### Methane and carbon dioxide flux measurements

Concentration changes of methane and carbon dioxide were measured using a GasScouter (G4301; PICARRO, Santa Clara, CA) with a static chamber on site. For terrestrial or shallow wetland sites, the chamber was placed on the surface of the soil/sediment or sedge/moss material, whereas the chamber was placed on the water surface for pond sites. Measurements were performed multiple times on each site. For each measurement, the molar fraction of methane and carbon dioxide was corrected based on effects of water vapor, and rates of concentration change were estimated over at least 60 s measurement time. Gas flux was estimated from the resulting rate.

### CARD-FISH analysis

Collected snow core samples were fixed in 4% paraformaldehyde in phosphate-buffered saline (PBS; 130 mM NaCl, 10 mM sodium phosphate, pH = 7.3) overnight at 4°C. Fixed samples were then washed twice in PBS and stored in a 1:1 mixture of PBS:ethanol at −20°C. Probes sequences and formamide concentrations, including for the Mγ669 probe used to target *Methylococcales* members, are described in the Supplementary Methods. An aliquot of fixed sample, mixed in a 1:1 ratio with 0.1% agarose, was spread on a gelatin-coated slide glass prepared according to [47]. Subsequent CARD-FISH preparation was performed according to [48, 49]. Cell walls were permeabilized by incubating the spread samples in 10 mg ml^−1^ lysozyme (dissolved in 0.1 M Tris–hydrochloric acid, 0.05 M EDTA, pH = 8.0) for 35 min at 37°C. Endogenous peroxidase was inactivated by incubation in a solution of 0.1 M hydrochloric acid at room temperature for 1 min, followed by washing with PBS for 1 min, incubation with 3% hydrogen peroxide for 10 min, and washing with PBS and water for 5 min and 1 min, respectively. CARD signal amplification was performed at 46°C using Alexa488-labeled and Alexa594-labeled tyramide. Visualization of samples was performed using a LSM780 laser scanning microscope (Zeiss, Jena, Germany) equipped with an Airyscan detector.

### Global sequence survey

The Sandpiper database, version 1.0.1 [50], was queried for meta-genomes containing members of the *Allocrenothrix* (i.e. UBA4132) genus, the *Crenothrix* genus, and the *Methylococcales* order. Only metagenomes having at least 0.01% relative abundances of *Allocrenothrix* or *Crenothrix* members were retained. In addition, metagenomes that seemed to be associated with laboratory cultures or synthetic microbial communities (based on “taxon”) were filtered from the analysis. The “taxon” entries for metagenomes were then summarized and collapsed into taxon groups to aid visualization (Supplementary Data 3).

## Results and discussion

### Isolation of filamentous methanotrophs

Sampling iron-rich mat material from the wetland at the sharp redox boundary between the oxic surface and anoxic underlying conditions allowed us to cultivate novel methanotrophs. Incubating the collected material under reduced O_2_ or anoxic conditions in a custom medium with methane and ferric iron, we observed growth of long filamentous bacteria that commonly associated with iron particles. Ferric iron could eventually be removed from the medium in later subcultures, and cultures that were started under anoxic conditions could be grown under 5% O_2_. After enrichment by filtration, micromanipulation, serial dilution, and multiple transfers to a refined selective medium (see Supplementary Methods), we obtained two independent axenic cultures of filamentous methanotrophic bacteria, named strains AF98 and MI19235. We validated the purity of the cultures via 16S rRNA gene amplicon sequencing (detection limit: 0.004%) and inoculation in dilute R2A medium.

### Methane-oxidizing physiology and cell biology

Previous attempts to establish cultures of filamentous methanotrophs have resulted in cultures with weak growth that could not be sustained beyond a few subcultures [51]. By contrast, strains AF98 and MI19235 exhibit robust growth as axenic cultures (Fig. 1, Fig. S1), with both strains having a 1–2 day doubling time and maximum observed culture densities of ~10^7^ cells ml^−1^. Axenic cultures have remained stable for more than one year of repeated subcultivation. When grown under 4% O_2_ with excess (17%) methane, strains uptake methane until O_2_ is depleted, with a O_2_:methane uptake ratio of approximately 1.0–1.4, i.e. well below the theoretical 2:1 stoichiometry of O_2_:methane in dissimilatory aerobic methane oxidation (Fig. 1A and B). The low O_2_:methane uptake ratio implies these strains supplement their methanotrophic metabolism with alternative metabolic pathways to conserve O_2_ [52]. The robust growth of these filamentous strains under methane and low O_2_ concentrations enabled study of their physiology and cell biology in the laboratory.

**Figure 1:**
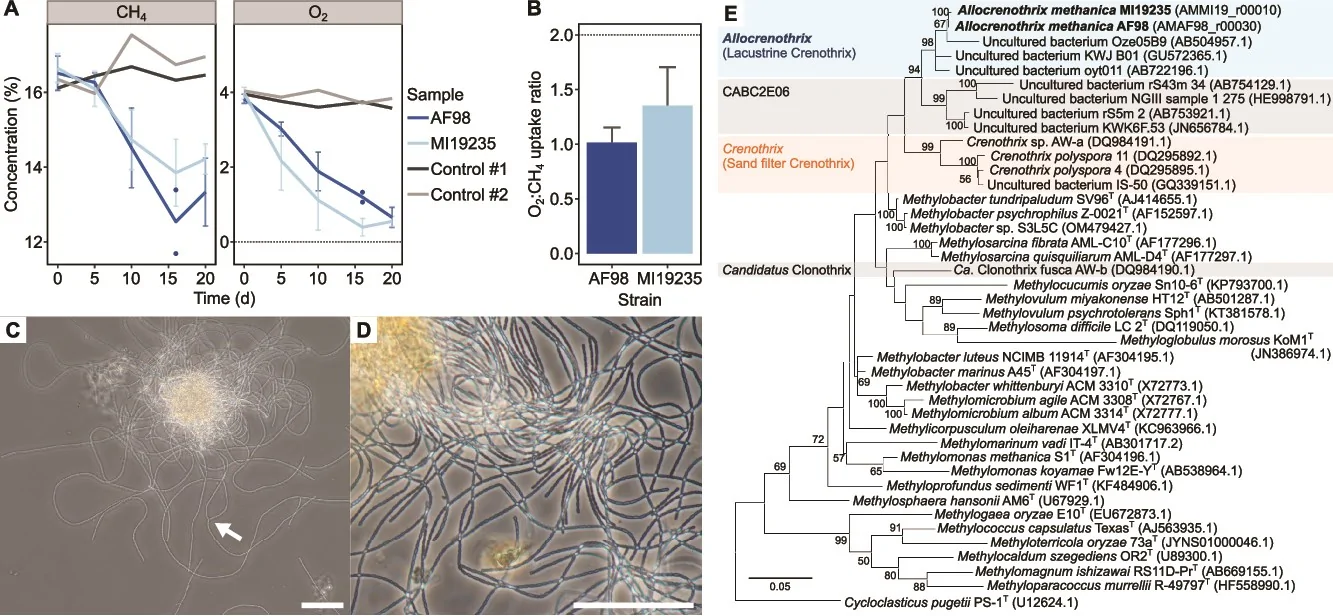
Cultivation of filamentous methanotrophs. (A) Oxidation of methane by strains AF98 and MI19235 compared to abiotic controls. Standard deviations of the mean are shown for biological replicates as error bars (*n* = 3). (B) O_2_:methane uptake ratios for cultures in (A) until complete O_2_ depletion (20 and 16 days for AF98 and MI19235, respectively; error bars are as in A). (C, D) Phase contrast microscopy images of strain AF98 grown under micro-oxic conditions with methane. The arrow (C) shows an example branching structure. The culture in (D) additionally included ferric iron particles. Scale bars represent 50 μm. (E) Maximum likelihood 16S rRNA gene phylogeny of members of the *Methylomonadaceae* family. The phylogeny was based on 1260 nucleotide sites with 100 bootstrap iterations. Bootstrap values over 50/100 are shown.

Most filaments of strains AF98 and MI19235 are linear and extend in length over the course of incubation, with filaments composed of rod-shaped cells of 1.0–1.3 × 1.9–4.3 μm for strain AF98 (Fig. 1C and D) and slightly larger cells of 1.1–1.5 × 2.5–5.0 μm for strain MI19235 (Fig. S1A). Whereas filaments of strain MI19235 are generally fragmented, filaments of strain AF98 can extend to hundreds of micrometers in length and occasionally develop branched structures, which we observe more frequently in fully grown cultures (Fig. 1C, Supplementary Videos 1–3). Tips of filaments are prone to adhesive attachment to surfaces or other filaments (e.g. Supplementary Video 3, bottom  left), and we commonly observe tight bundles of filaments for strain AF98 during growth (Fig. 1C). *Crenothrix polyspora* has been observed to produce spore-like structures called gonidia [16, 53], but we do not observe such structures for strains AF98 and MI19235. We also have not observed gliding motility for either strain, although we did observe a gradual serpentine motion of filaments that appeared distinct from Brownian motion of surrounding particles (Supplementary Videos 1–3). We confirmed based on 16S rRNA gene sequences that strains AF98 and MI19235 are members of the gammaproteobacterial *Methylococcales* order, which includes Crenothrix bacteria and other aerobic methanotrophs (Fig. 1E; see below).

At the sub-cellular level (Fig. 2) inside filaments (Fig. 2A, Fig. S1B), we observed folded intracytoplasmic membrane stacks that are typical for aerobic methanotrophs via transmission electron microscopy (Fig. 2B and C, Fig. S1C). We did not observe gas vacuoles, which may be used for buoyancy by some free-living methanotrophs [9]. Between cells in filaments, we identified unusual conjugation structures, where cell membranes apparently open directly into adjacent cells (Fig. 2D and E, Fig. S1C). These conjugations always occurred in a single directional orientation in filaments and were accompanied by a veil-like structure enrobing the connection (Fig. 2B, D and E). Less frequently, we also observed such conjugations on the long sides of cells, potentially resulting in cell pairs or branching filaments (Fig. S1D and E). Cytoplasmic bridges were consistently over 100 nm in diameter (Fig. 2E), implying they could facilitate movement of cytoplasmic contents between cells. Together, our observations clearly establish strains AF98 and MI19235 as filamentous aerobic methanotrophs capable of growth under low O_2_, with a lack of gonidia and presence of unique cellular structures implying these strains represent a distinct form of Crenothrix bacteria compared to those reported previously.

**Figure 2:**
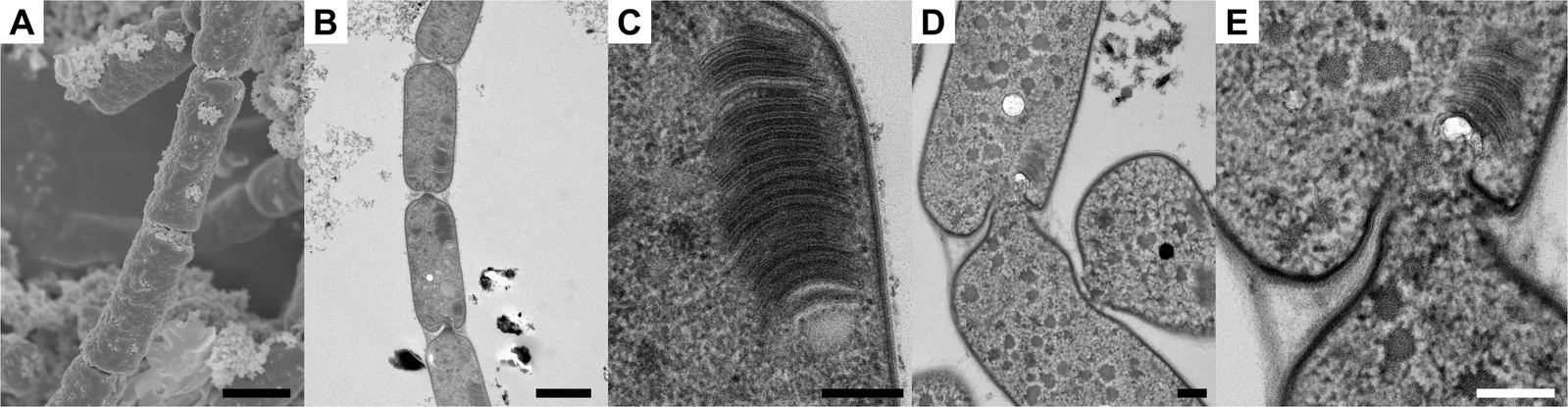
Sub-cellular physiology of methanotroph filaments. Images of strain AF98 are shown when grown under micro-oxic conditions with methane and ferric iron particles (see Fig. S1 for images of strain MI19235). (A) Scanning electron microscopy image of a representative cellular filament. (B, C) Transmission electron microscopy images of connected cells in a filament (B) and their internal structures (C; image taken from the center area of B). (D, E) Transmission electron microscopy image of a cell-to-cell conjugation (E is taken from the center area of D). Scale bars represent 1 μm (A–B) and 0.2 μm (C–E).

### Phylogenetic placement

We sequenced and annotated closed circular genomes of strains AF98 and MI19235 to understand their evolutionary placement (Fig. 3). The two genomes had total lengths of 4 134 542 bp and 4 298 823 bp for AF98 and MI19235, respectively, and consisted of single circular chromosomes and several circular plasmids (eight for AF98 and five for MI19235). Each strain has three identical 16S rRNA gene copies per chromosome (Supplementary Data 1). Known Crenothrix bacteria can be subdivided into multiple polyphyletic clades within the *Methylomonadaceae* family of the *Methylococcales* order [54]. These include the “sand filter Crenothrix” clade containing *C. polyspora* and a “lacustrine Crenothrix” clade containing filamentous methanotrophs observed in stratified lakes [14]. Previous studies have also reported a third clade represented by the filamentous methanotroph *Clonothrix fusca*, which was available in a poorly growing enrichment culture but has not been reported on in nearly 20 years [51, 55]. Based on a 16S rRNA gene phylogeny (Fig. 1E, Fig. S2) and concatenated protein phylogeny (Fig. 3A), strains AF98 and MI19235 fall into the lacustrine Crenothrix clade alongside the uncultured *Crenothrix* sp. D3 [14]. Classification into the lacustrine Crenothrix clade is supported by classification according to the GTDB (release 220), which places strains AF98 and MI19235 in the uncultured UBA4132 genus represented by strain D3 [31].

**Figure 3:**
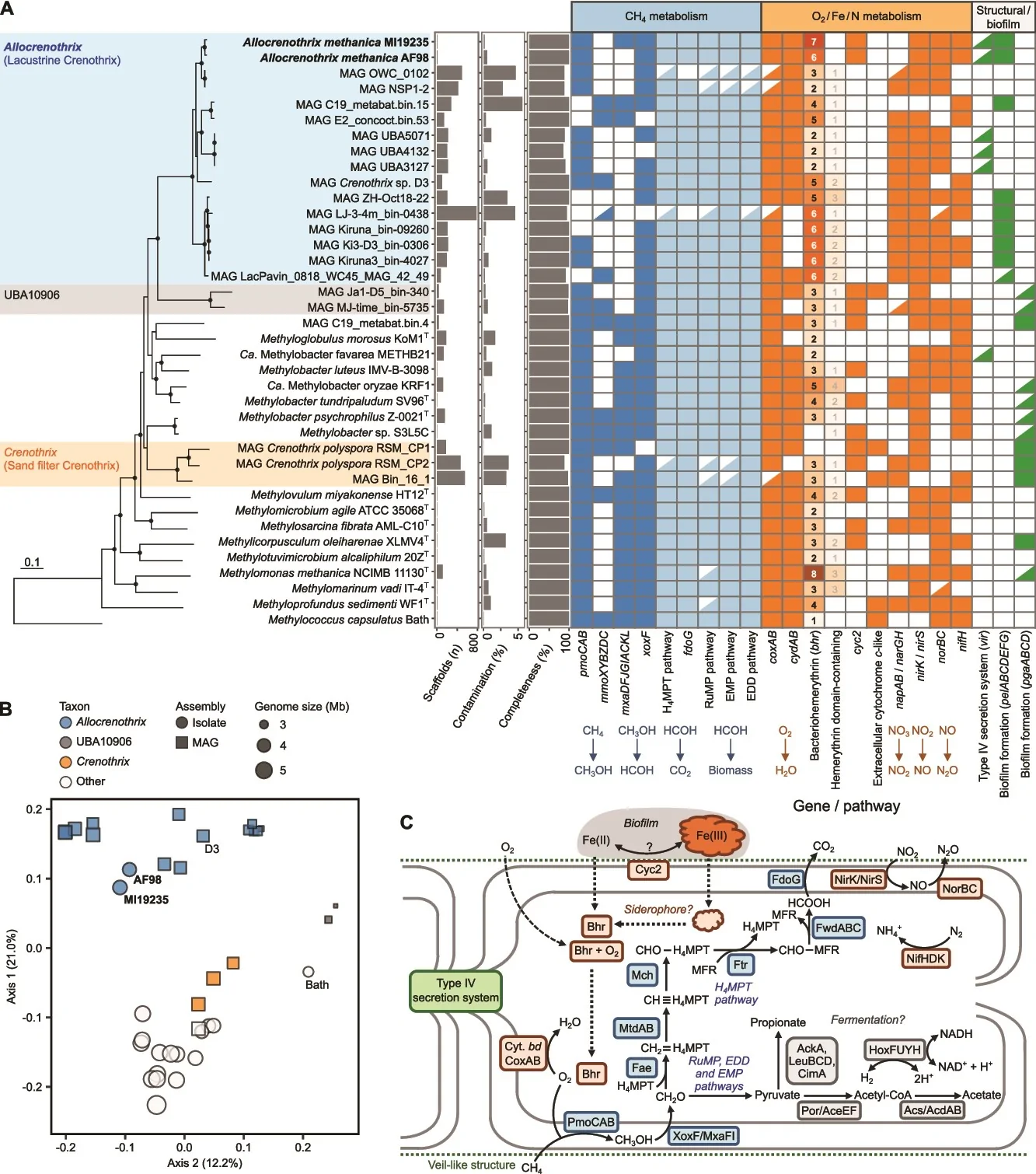
Genomic characterization of *Allocrenothrix* members. (A) Comparative genomic analysis of members of the *Methylomonadaceae* family. The maximum likelihood phylogeny (left) was constructed based on 170 concatenated marker proteins with 1000 ultrafast bootstraps. Bootstrap values over 90/100 are shown as black dots. Contamination and completeness statistics (center) are based on CheckM2. In the gene heatmap (right), solid squares indicate presence of a gene/pathway, solid triangles indicate presence of over half the genes in a pathway, and open squares indicate absence of a gene or presence of less than half the genes in a pathway. Numbers on solid squares for (bacterio)hemerythrin-related genes, along with the intensity of shading, indicates gene copy number. Additional genome statistics and locus information for detected genes are shown in Supplementary Data 1. (B) Ordination of genomes in (A) based on counts of orthologous gene groups in each genome. (C) Cell diagram showing the proposed metabolism of strains AF98 and MI19235.

Strains AF98 and MI19235 share an average nucleotide identity of 95.3% and a 16S rRNA gene sequence identity of 99.8%. This, together with their related physiologies, leads us to propose them as two distinct phylotypes of the same novel lacustrine Crenothrix species [56]. For reasons of priority, we maintained the name *Crenothrix* for the genus described for Cohn’s *C. polyspora* and therefore named AF98 and MI19235 as strains of *Allocrenothrix methanica* (Allocrenothrix, i.e. “other Crenothrix”; see etymology below), with the GTDB clade UBA4132 (i.e. lacustrine Crenothrix) now becoming the genus *Allocrenothrix*. The species *Allocrenothrix methanica* was registered in the SeqCode database using the genome of strain AF98 as a voucher. The proposed type strain AF98 and strain MI19235 are currently being prepared for deposition into two culture collections, so the genus and species names retain *Candidatus* status under the International Code of Nomenclature of Prokaryotes.

### Comparative genomics

The physiology of classical *Crenothrix* (i.e. sand filter Crenothrix) and *Clonothrix* species have been studied to some degree in the laboratory [18, 51, 53], but the lacustrine Crenothrix clade (i.e. *Allocrenothrix*) has not been characterized in depth beyond environmental genomics and microscopy. We therefore performed comparative genome analyses of *Allocrenothrix methanica* strains AF98 and MI19235 against other cultured and uncultured potential methanotrophs. Our analyses focused on members of the *Methylomonadaceae* family, which encompasses all known Crenothrix bacteria. We found that the AF98 and MI19235 genomes, along with most metagenome-assembled genomes (MAGs) classified as *Allocrenothrix*, encode *pmoCAB* genes for a particulate methane monooxygenase along with genes for the classical pathway of aerobic methane oxidation (Fig. 3A and Note S1; all genes are listed by locus in Supplementary Data 1). At the same time, in place of the *mxaF* gene for calcium-dependent methanol dehydrogenase, many *Allocrenothrix* members encode the *xoxF* gene for the lanthanide-dependent enzyme [57] (Fig. S3), and we speculate that dependence on lanthanides could be one reason for previous challenges in cultivating these filamentous methanotrophs.

*Allocrenothrix* members commonly have ample genomic potential for addressing O_2_ limitation. Along with genomic potential for aerobic respiration, including the *coxAB* genes encoding a *caa*3-type cytochrome *c* [58, 59], the majority of *Allocrenothrix* members (including AF98 and MI19235) encode at least five homologs of bacteriohemerythrin, a non-heme-based O_2_ binding protein implicated in O_2_ storage in gammaproteobacterial methanotrophs [60–64] (Fig. 3A, Table S2). By contrast, the genomes we analyzed of cultured unicellular *Methylomonadaceae* members generally encode three or fewer of these genes. These analyses were robust when extended to a representative set of over 300 genomes/MAGs from the *Methylococcales* order, with *Allocrenothrix* members having a median hemerythrin domain-containing protein count of eight compared to a median of 1–4 in other analyzed *Methylococcales* lineages (Table S2). The majority of analyzed *Allocrenothrix* members, including strains AF98 and MI19235, also encode genes for partial denitrification of nitrite to nitrous oxide, including a *nirK* or *nirS* homolog for nitrite reductase and *norBC* homologs for nitric oxide reductase [65] (Fig. 3A). Some *Allocrenothrix* members (not AF98/MI19235) additionally encode *narGHI* homologs for nitrate reductase. Although denitrification genes are commonly encoded by isolated unicellular *Methylomonadaceae* members, the gene pathway is absent in all MAGs of classical *Crenothrix* members (Fig. 3A, Table S2). The genomes of strains AF98 and MI19235 encode potential pathways for fermentation of pyruvate to acetate and/or propionate (Note S1). In addition, homologs of the “cluster 2” form of *cyc2*, which has been implicated in extracellular electron transfer in some iron-oxidizing bacteria [66], were detected in both genomes (Note S1). The metabolic role of *cyc2* remains poorly explored [67], but if iron reduction via extracellular electron transfer is possible by these strains, then this metabolism could further supplement their O_2_ requirements. Extracellular electron transfer could additionally be used to balance the redox conditions in cells of the strains [68] or to modulate the redox state of extracellular iron, whether to control the physical scaffolding around filaments or regulate uptake of different iron forms in iron-rich and O_2_-limited environments (e.g. Fig. 3).

To explore genes potentially unique to filamentous methanotrophs, we performed a pan-core analysis of *Allocrenothrix*, its sister lineage UBA10906, *Crenothrix*, and other members of the *Methylomonadaceae* family (Fig. S4). When separated in an ordination based on counts of orthologous gene groups in genomes, members of *Allocrenothrix* formed a separate cluster from other clades (PERMANOVA, *n* = 38, *F* = 6.2, *P* = .001), which further supports that the *Allocrenothrix* lineage is genomically distinct from its sibling lineages (Fig. 3B, Fig. S4A). Aside from core genes common to *Methylomonadaceae* members, <4% of orthologous gene groups were shared between *Allocrenothrix* and *Crenothrix* members, and none of these genes had clear roles related to cell division or cell structure based on annotation data (Fig. S4B and C; genes are summarized in Supplementary Data 1). Given their phylogenetic separation and unique functional gene profiles, it is possible that *Crenothrix* and *Allocrenothrix* were shaped by distinct evolutionary pressures and could have even evolved filamentous morphology independently.

Among orthologous gene groups that were unique to *Allocrenothrix* members, we detected a *pelABCDEFG* gene cluster, associated with biosynthesis of extracellular polysaccharides involved in biofilm formation [69–73], that was conserved in strain AF98, MI19235, and several other members of the genus (Fig. 3A). This was distinct from the *pgaABCD* genes implicated in biofilm formation that we detected in *Crenothrix* members and other methanotrophs (Fig. 3A). Beyond this, we detected a cluster of *vir* genes, encoding a Type IV secretion system, that were encoded by several *Allocrenothrix* members including strains AF98 and MI19235 (Fig. 3A, Note S1). We speculate that production of extracellular polysaccharides may be important for scaffolding and attachment of filamentous cells, e.g. allowing cells to remain at locations with optimal concentrations of O_2_ and methane, and Type IV secretion systems could potentially play a role in the cell conjugations we observed (e.g. Fig. 3C) [74]. Together, our analyses establish *Allocrenothrix* as a genomically distinct lineage of aerobic methanotrophs with enhanced genomic potential for coping with O_2_ limitation compared to related clades.

### Detection in iron-rich wetlands

To clarify the potential ecologies of *Allocrenothrix* members, we surveyed the subalpine wetlands from which strains AF98 and MI19235 were isolated and performed amplicon sequencing, microscopy-based analyses, and gas flux measurements (Fig. 4). Along with non-iron rich peatlands, the wetlands contain abundant iron-rich zones that can be subdivided into extreme iron-rich mats from which both strains were cultured, iron-rich mat residue mixed with plant material, and iron-rich snow material that forms over iron mat regions in winter (Fig. S5A–C). Iron-rich snow regions can be temporally distinguished as pre-melt material and snow in the final stages of spring melt, where a reddish precipitate appears on the snow surface in a phenomenon locally called “Akashibo” [75]. Iron-rich mats and their overlying snow were commonly stained an intense reddish-brown color and could develop high dissolved iron concentrations, which reached up to 0.3 mM in surficial mat samples, highlighting the potentially steep redox gradients between oxic and anoxic wetland zones in these regions (Supplementary Data 2; see also [75, 76]).

**Figure 4:**
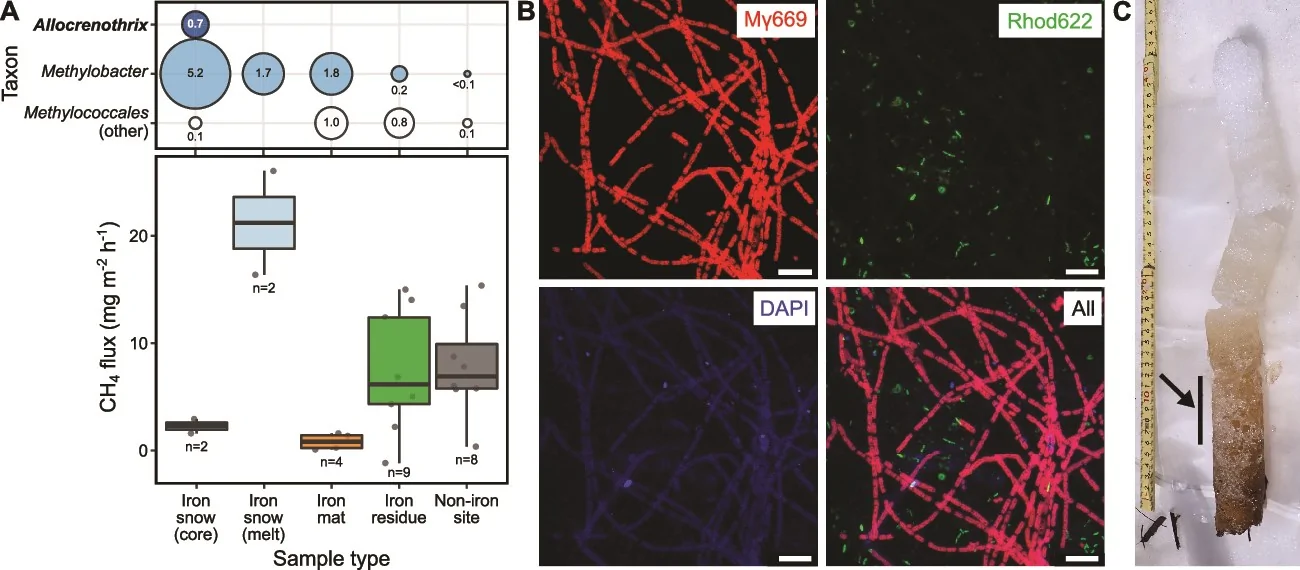
Ecology of filamentous methanotrophs in iron-rich wetlands. (A) Gas fluxes and potential methanotroph communities from wetland samples. The bubble plot (top) shows the maximum relative abundances of *Methylococcales* clades detected from each wetland site type based on 16S rRNA gene amplicon data. The boxplots (bottom) show the methane fluxes measured from distinct wetland sites and seasonal timepoints within each site type. Amplicon and flux data per site/timepoint are shown in Fig. S6, and raw data are available in Supplementary Data 2. (B) Microscopy image of iron-rich snow core material (from site KOJ20, 6–12 cm above surface; see below) using the CARD-FISH assay. Results are shown for the Type I methanotroph probe Mγ669 (top left), DAPI staining (bottom left), and the Rhod622 probe targeting *Rhodoferax* members as a control (top right). The data are also shown overlaid (bottom right). Scale bars represent 10 μm. Additional CARD-FISH images are shown in Fig. S8. (C) Iron-rich snow core from the KOJ20 site. The arrow indicates the snow core region used for CARD-FISH analyses shown in (B).

Collecting samples across multiple seasons and years, we observed large differences in methane fluxes (Fig. 4A and Fig. S6). Iron-rich snow cores (2.9 and 1.6 mg methane m^−2^ h^−1^, *n* = 2) and iron mat zones (0.83 mg methane m^−2^ h^−1^ ± 0.76 S.D., *n* = 4) had consistently low measured methane fluxes despite overlying anoxic sediments, whereas iron residue (7.21 mg methane m^−2^ h^−1^ ± 5.52 S.D., *n* = 9), and non-iron rich sites (7.91 mg methane m^−2^ h^−1^ ± 4.73 S.D., *n* = 8) had higher but variable methane fluxes. Zones in the final stages of snow melt had the highest observed methane fluxes (16.4 and 26.1 mg methane m^−2^ h^−1^, *n* = 2), potentially due to rapid methane release during spring thaw or disruption of microbial communities. Differences between wetland site types were evident at the microbial community level, with an ordination of 16S rRNA gene amplicon data (Fig. S7) indicating that iron-rich snow, iron mat, iron residue, and non-iron rich samples formed statistically robust groupings (PERMANOVA, *n* = 25, *F* = 3.3, *P* = .0001). Along with lower methane emissions, iron-rich snow core and iron mat samples also had elevated relative abundances of *Methylococcales* members (3.06% ± 2.05 S.D., *n* = 6), based on amplicon data, compared to samples from other sites (0.24% ± 0.48 S.D., *n* = 16; Mann–Whitney *U* = 93.0, *P* = .00057, two-sided). Together, these gas flux and amplicon data suggest that iron-rich snow and iron mat zones could be key sites for aerobic methanotrophy in the wetland.

Within the detected *Methylococcales* sequences, we identified *Allocrenothrix* members based on manual reclassification of amplicon data by phylogenetic placement (Fig. 4A; see Supplementary Methods). Although we did not detect *Allocrenothrix* members in iron-rich mat samples from which strains AF98 and MI19235 were isolated (detection limit: 0.007%), we identified multiple *Allocrenothrix* sequence variants from snow core samples, including one sequence variant that exactly matched the sequences of strains AF98 and MI19235 and had relative abundances of up to 0.6% (sequence oze-d3cbca2; see Supplementary Data 2). Sequences of *Allocrenothrix* members were also identified from an iron-rich snow sample that was collected from the wetland over 15 years ago (GenBank AB504957.1; Fig. 1E) [75], which suggests that *Allocrenothrix* members have long-term environmental relevance within the methanotroph communities in this region. We always detected other *Methylococcales* members in samples with *Allocrenothrix* sequences, and *Allocrenothrix* members made up ~8%–15% of the potential gammaproteobacterial methanotroph community (Fig. S6). By contrast, we did not detect classical *Crenothrix* members in any sample analyzed.

We performed microscopic single cell identification and quantification of iron-rich snow core material using CARD-FISH (Fig. 4B and C). By staining with the *Methylococcales* probe Mγ669, whose sequence exactly matches that of strains AF98 and MI19235 except for a single base on the 3′ end, we observed long Mγ669-stained filaments that likely represent *Allocrenothrix* members along with free-living Mγ669-stained cells (Fig. 4B–C, Fig. S8A and B). The filamentous arrangements of the visualized cells were analogous to transmission electron microscopy observations of strains AF98 and MI19235. Samples were heterogeneous, and the Mγ669-stained cells varied in their morphology. Yet filamentous methanotrophs consistently appeared to be important members of the *Methylococcales* in these iron-rich snow samples, potentially thriving with unicellular methanotrophs on dissolved compounds and gasses diffusing from the underlying iron mat regions. That strains AF98 and MI19235 exactly match the dominant *Allocrenothrix* sequence variant from these systems suggests that adaptations observed in these strains are representative of those employed by filamentous methanotrophs in the environment.

### Global ecology

We surveyed publicly available metagenome data to understand the broader distribution of *Allocrenothrix* members (Fig. 5). Searches for classical *Crenothrix* members were used as a baseline for our analyses. Querying over 700 000 metagenome samples via the Sandpiper database [50], we uncovered 1427 samples that included *Allocrenothrix* (i.e. UBA4132) members, compared to 156 samples for *Crenothrix* members (Fig. 5A). Comparing the environmental (“taxon”) attributions of metagenomes from which these genera were identified, we found that members of both genera were commonly detected in freshwater samples, but *Allocrenothrix* members were more frequently associated with sediment, soil, wastewater/sludge, and peat samples than *Crenothrix* members (Fig. 5B; Supplementary Data 3). By contrast, *Crenothrix* members were more frequently associated with biofilm, subsurface/groundwater, aquaculture/biofilm, and drinking water samples (Fig. 5B). Despite known accuracy limitations of “taxon” attributions of metagenomes, results for *Crenothrix* members match literature reports of these bacteria in oligotrophic environments such as drinking water and groundwater [16, 17], implying these data capture real trends. Widespread detection of *Allocrenothrix* members in environments that typically include redox gradients, such as sediments and sludges, is consistent with genomic and field survey data that indicate these organisms commonly encounter O_2_ limitation.

**Figure 5:**
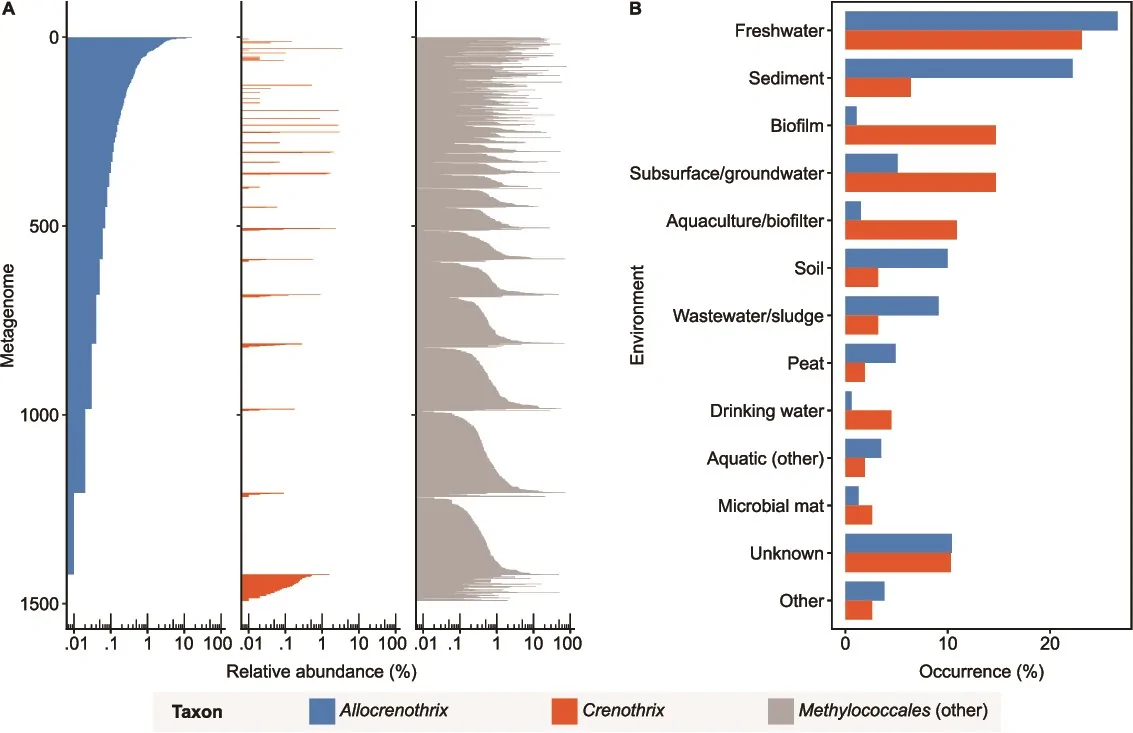
Global distribution of potential filamentous methanotrophs. (A) Relative abundances of *Allocrenothrix* and classical *Crenothrix* members in publicly available metagenome samples. Samples containing at least 0.01% of members of these genera are shown. (B) Environmental affiliations (by “taxon”) of metagenomes where *Allocrenothrix* and *Crenothrix* members were detected. Taxa are collapsed into higher-level environmental groups (see Supplementary Data 3), and groups are shown if they included at least 1.5% of metagenomes where a methanotroph taxon was detected. Other groups are collapsed as “Other”.

### Ecophysiology linked to O_2_ limitation

Despite their observation over 150 years ago, filamentous methanotrophs have remained severely understudied in their ecological roles due to a lack of available laboratory cultures. Our isolation of two filamentous methanotroph strains and analysis of their physiology and complete genome sequences provide fundamental new insights into the ecophysiology of this elusive bacterial group. Given that *Allocrenothrix methanica* strains AF98 and MI19235 grow at reduced O_2_ with low O_2_:methane uptake ratios, that these strains encode high numbers of genes linked to O_2_ limitation compared to methanotrophs in related clades, and that close relatives of these strains are detected in methane-containing and potentially reducing environments, our data strongly indicate that *Allocrenothrix methanica* and other species in the *Allocrenothrix* genus are highly adapted to life under O_2_ limitation. These results match previous detection of *Allocrenothrix* (i.e. lacustrine Crenothrix) members in stratified lake systems [14]. Moreover, our results indicate that large genomic differences exist between filamentous methanotroph groups, with classical *Crenothrix* (i.e. sand filter Crenothrix) members potentially having distinct evolutionary adaptations and ecological preferences from the *Allocrenothrix* group.

Although the mechanisms driving the O_2_ limitation-linked ecophysiology of *Allocrenothrix* remain to be explored in detail, our cell biology observations and genomic findings allow us to propose potential adaptations that may allow these filamentous methanotrophs to inhabit low O_2_ environments (Fig. 6). Although strains AF98 and MI19235 do not possess gliding motility, they are prone to surface attachment and have relatively large cytoplasmic volumes compared to unicellular methanotrophs, as observed for *Allocrenothrix* members in the environment [14]. Rather than relying on motility like certain unicellular methanotrophs, we thus propose that *Allocrenothrix* members could anchor via biofilms at regions with optimal methane and O_2_ concentrations, and cells could then use stored compounds, such as O_2_-bound bacteriohemerythrins, to persist under periods of transient redox change such as O_2_ depletion (Fig. 6A). Strains AF98 and MI19235 also possess unique cellular connective structures, and this leads us to speculate that these structures play a role in the movement of compounds between cells in a filament. Sharing of small metabolic intermediates such as methanol [77], produced during the O_2_-dependent step of aerobic methane oxidation, between connected cells across redox gradients could potentially supply O_2_-deficient cells with carbon and energy sources in a manner somewhat analogous to electron transport by cable bacteria (Fig. 6B) [23, 24]. These proposed models to “store” or “share” metabolic intermediates could potentially enhance both persistence and activity of *Allocrenothrix* cells in O_2_-limited niches and could provide competitive advantages compared to unicellular methanotrophs. With cultures of strain AF98 and MI19235 now available for study, these models and their implications for global methane cycling can now be explored in detail in future work.

**Figure 6:**
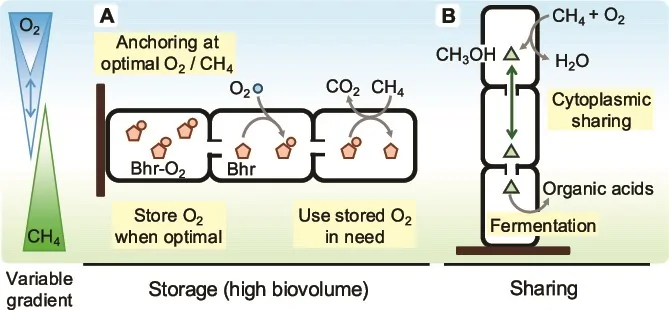
Proposed strategies of filamentous *Allocrenothrix* members to address O_2_ limitation. The diagram (not to scale) includes an example O_2_:methane counter-gradient on the left with variability in the steepness of the redox gradient (e.g. O_2_ availability). (A) Cells in filaments may rely on their high biovolumes for storage of metabolic intermediates to withstand transient redox change. (B) In addition, connected cells may share metabolites to remain active across micro-scale redox gradients.

## Conclusion and outlook

Ever since the seminal discovery of the “Brunnenfaden” by Ferdinand Cohn, microbiologists have gradually illuminated the function and ecology of Crenothrix bacteria in the global environment. The isolation of two strains of lacustrine Crenothrix represents a critical next step for understanding the basis of the methanotrophic metabolisms of these bacteria, the nature of their cellular interactions, and the adaptations that govern their ecological preferences compared to unicellular and free-living strains. Probing the lifestyle of *Allocrenothrix methanica* in particular could yield important insights into how methanotrophs adapt to seemingly anoxic ecosystems where they are commonly detected [9]. Indeed, the potential for methanotroph filaments to behave as a multicellular organism may shape the O_2_ responses of these bacteria. Furthermore, a potential link between methane cycling and iron metabolism merits further exploration. The two stable, axenic cultures of *Allocrenothrix methanica* we present are readily available for study and open vast new opportunities for research on this enigmatic group of bacteria.

## Species description

*Allocrenothrix methani*ca

**Etymology.**  Al.lo.cre.no.thrix. Gr. masc. pron. *allos*, other, another, different; N.L. fem. n. *Crenothrix*, a bacterial genus; N.L. fem. n. *Allocrenothrix*, the other *Crenothrix*. Me.tha.ni.ca. N.L. neut. n. *methanum*, methane; L. fem. adj. suff. -*ica*, pertaining to; N.L. fem. adj. *methanica*, pertaining to methane.

**Locality.** Isolated from iron-rich wetland sediments collected from Oze National Park, Japan.

**Diagnosis.** Aerobic methane oxidizing-bacterium of the “lacustrine Crenothrix” clade. Grows in linear, rarely branched filaments of cells around 1.0–1.5 μm wide and 1.9–5.0 μm long with no production of gonidia. Cells are connected by conjugation-like structures. There are two strains AF98 and MI19235 for which closed genomes are available (GenBank accession numbers AP041007-AP041015 and AP041016-AP041021, respectively), with AF98 being the proposed type strain. Strain AF98 is deposited in the SeqCode as strain sc|0040749. Both strains are available in the German Collection of Microorganisms and Cell Cultures under strain numbers DSM 122070 (AF98) and DSM 122071 (MI19235).

## Supplementary Material

Supplementary_material_wrag178

## Data Availability

The complete genomes of strains AF98 and MI19235 are available at the DNA Data Bank of Japan (DDBJ) at nucleotide accessions AP041007-AP041015 and AP041016-AP041021, respectively and are summarized under DDBJ BioProject PRJDB20581. Raw short-read (DNBSEQ-based) genome sequence data are available from DDBJ run accessions DRR905474 and DRR905476 for strains AF98 and MI19235, respectively. In addition, long-read (Nanopore-based) genome sequence data are available for strain AF98 from DDBJ run accessions DRR905473, DRR905477, and DRR905478, and for strain MI19235 from accessions DRR905475, DRR905479, and DRR905480. Raw (Illumina-based) 16S rRNA gene amplicon data for pure culture samples of strains AF98 and MI19235 are available at DDBJ run accessions DRR905485 and DRR905486, respectively. In addition, raw (Illumina-based) 16S rRNA gene amplicon data for wetland samples are summarized at DDBJ BioProject PRJDB40312 and are available at DDBJ run accessions DRR906888 to DRR906912, as described in detail in Supplementary Data 2. *Allocrenothrix methanica* strains AF98 and MI19235 are currently available through the German Collection of Microorganisms and Cell Cultures under strain numbers DSM 122070 and DSM 122071, respectively.
